# Implications of Trauma among Male and Female Offenders

**DOI:** 10.3390/ijerph9010097

**Published:** 2012-01-03

**Authors:** Flora I. Matheson

**Affiliations:** Centre for Research on Inner City Health, The Keenan Research Centre in the Li Ka Shing Knowledge Institute of St. Michael’s Hospital, 30 Bond Street, Toronto, Ontario M5B 1W8, Canada; Email: mathesonf@smh.ca; Tel.: +1-416-864-6060 (etx. 77482); Fax: +1-416-864-5485

Criminal behaviour is believed to arise from a multiplicity of factors, including unemployment and poverty [1,2], low self-control [3], psychological issues [4,5], early conduct problems [6], childhood physical and sexual abuse disorder [5], and social bonding in child- and adulthood [7]. Social-structural influences like family conflict/disruption, financial resources, child-parent and school/peer attachment and abuse and neglect in childhood have lasting impressions, leading to multiple problems including delinquency and later criminal activity, substance use/abuse, mental illness and poor self-rated health [8,9,10,11,12]. The consequences of such behaviour include financial losses, injury, and death that together have significant personal and societal costs. Society also bears the burden of incarcerating and rehabilitating offenders; a burden that is not trivial. Direct costs of imprisonment in Canada approach $3.5 billion annually; in the US the cost is substantially higher, approaching $74 billion [13].

Adolescent delinquency, adult criminal behavior, mental health and addictions, and self-harm are all repercussions of the recognized physical and sexual abuses that girls and women are often subjected to during youth and adolescence and into the adult years. Physical and sexual abuse of boys is less documented. Historically, men have been the focus on research in sociology, criminology, medicine and epidemiology to the exclusion of women until the research community began to understand that gender does complicate matters. Yet, there remains scarce information on the repercussions of sexual and physical trauma of boys, despite considerable media coverage of indiscretions within organizations heavily focused on boys (e.g., the church, residential schools, and sports teams). This has perpetuated a societal belief that the problem is not common and the outcomes are not as severe among boys. The reality is very different. While depression and anxiety are not considered the domain of boys and men, reports of suicide of boys who have been sexually abused speak to the devastation of trauma [14,15]. Holmes *et al.* [16] conducted a systematic review of the literature on sexual abuse of boys. They found that the literature was sparse and highly differentiated with respect to methodology and definitions; sampling techniques were poor; and, few studies controlled for effect modifiers. The study comments on the sequelae of abuse among boys: psychological distress, substance abuse and sexually-related problems (e.g., dysfunction, hypersexuality, sexually aggressive behavior and confused sexual identity). A comprehensive literature review in 2002 included 62 studies comprising 23,000 prisoners and concluded that 1 in 7 prisoners have a psychotic illness or major depression; and, 1 in 2 male and 1 in 5 female prisoners have antisocial personality disorder. Post-traumatic stress disorder (PTSD) was not explored in any of the studies, despite growing evidence that trauma is highly prevalent in this population [17].

Information on the prevalence and severity of lifetime interpersonal violence encountered by imprisoned men and women is scarce, often contradictory and subject to under-reporting. Actual prevalence rates vary depending on the scope of traumatic events examined. Estimates range from a low of 13% to in excess of 75% of offenders who have experienced childhood physical or sexual abuse [17,18,19]. Estimates for women prisoners are often twice as high as among males [20]. Male and female prisoners report twice the rate of the general population [20].

The consequences of trauma persist long after the experience is over and produce lifelong problems like depression, PTSD, substance abuse, low occupational attainment, poor medical health, and criminal involvement. With the growth in the prison population in the US and now with new laws in Canada (the Safe Streets and Communities Act and the Truth in Sentencing Act [21]) the prison population will swell over the coming years. 

Given that the majority of prisoners have experienced trauma, there is a need to examine current interventions for trauma that might prove effective for male and female offenders. Research has shown that trauma-informed care—the focus of which is on understanding how to work with a woman regardless of where she is in the recovery cycle—is an important feature to include in treatment targeting substance abuse [22,23]. Holistic approaches with a gendered focus are proffered as most effective for women given their multiple health needs (e.g., trauma, mental illness, substance abuse). But, such approaches are also relevant for male offenders who are struggling with very similar health-related problems. Whether these modified intervention approaches (those which also address trauma) will look different for men and women is not clear at this point. What is clear is that there is a need for treatment that addresses trauma in this highly vulnerable population. 

This special issue of the *International Journal of Environmental Research and Public Health* provides a venue to shed light on the pervasiveness of trauma in the criminal justice system; explore the personal, social and health consequences of trauma for offenders; and, ponder what treatments might prove effective in this particularly vulnerable population.

## References

[1] Avakame E.F. (1997). Urban homicide: A multilevel analysis across Chicago’s census tracts. Homicide Stud..

[2] Kawachi I., Kennedy B.P., Wilkinson R.G. (1999). Crime: Social disorganization and relative deprivation. Soc. Sci. Med..

[3] Gottfredson M.R., Hirschi T. (1990). A General Theory of Crime.

[4] Sutker P.B., Moan C.E. (1973). Prediction of socially maladaptive behavior within a state prison system. J. Community Psychol..

[5] Caspi A. (2000). The child is father of the man: Personality continuities from childhood to adulthood. J. Personal. Soc. Psychol..

[6] Sampson R.J., Laub J.H. (1990). Crime and deviance over the life course: The salience of adult social bonds. Am. Sociol. Rev..

[7] Laub J.H., Sampson R.J. (1993). Turning points in the life course: Why change matters to the study of crime. Criminology.

[8] Delaney-Black V., Covington C., Ondersma S.J., Nordstrom-Klee B., Templin T., Ager J., Janisse J., Sokol R.J. (2002). Violence exposure, trauma, and IQ and/or reading deficits among urban children. Arch. Pediatr. Adolesc. Med..

[9] Schwartz D., Proctor L.J. (2000). Community violence exposure and children’s social adjustment in the school peer group: The mediating roles of emotion regulation and social cognition. J. Consult. Clin. Psychol..

[10] Widom C.S., Ames M.A. (1994). Criminal consequences of childhood sexual victimization. Child Abuse and Neglect. The International Journal.

[11] Widom C.S., Weiler B.L., Cottler L.B. (1999). Childhood victimization and drug abuse: A comparison of prospective and retrospective findings. J. Consult. Clin. Psychol..

[12] Widom C.S., Marmorstein N.R., White H.R. (2006). Childhood victimization and illicit drug use in middle adulthood. Psychol. Addict. Behav..

[13] Tracey K. (2007). Justice Expenditure and Employment Extracts.

[14] Molnar B.E., Shade S.B., Kral A.H., Booth R.E., Watters J.K. (1998). Suicidal behavior and sexual/physical abuse among street youth. Child Abuse Negl..

[15] Martin G., Bergen H.A., Richardson A.S., Roeger L., Allison S. (2004). Sexual abuse and suicidality: Gender differences in a large community sample of adolescents. Child Abuse Negl..

[16] Holmes W.C., Slap G.B. (1998). Sexual abuse of boys. J. Am. Med. Assoc..

[17] Weeks R., Widom C. (1998). Self-reports of early childhood victimization among incarcerated adult male felons. J. Interpers. Violence.

[18] Dutton D.G., Hart S.D. (1992). Evidence for long-term, specific effects of childhood abuse and neglect on criminal behavior in men. Int. J. Offender Ther. Comp. Criminol..

[19] Lewis D.O., Shanok S.S., Pincus J.H., Glaser G.H. (1979). Violent juvenile delinquents: Psychiatric, neurological, psychological, and abuse factors. J. Am. Acad. Child Psychiatry.

[20] (1999). Prior Abuse Reported by Inmates and Probationers.

[21] The Safe Streets and Communities Act is an act to enact the Justice for Victims of Terrorism Act and to amend the State Immunity Act, The Criminal Code, the Controlled Drugs and Substances Act, the Corrections and Conditional Release Act, the Youth Criminal Justice Act, the Immigration and Refugee Protection Act and Other Acts. The Truth in Sentencing Act is designed to limit the credit for time spent in pre-sentencing custody (“credit for time served”). http://www.parl.gc.ca/Default.aspx?Language=E.

[22] Herman J.L. (1992). Trauma and Recovery.

[23] Bloom B. (1999). Gender-responsive programming for women offenders: Guiding principles and practices. Forum on Corrections Research.

